# Factors affecting the catch-up growth of preterm infants after discharge in China: a multicenter study based on the health belief model

**DOI:** 10.1186/s13052-019-0674-2

**Published:** 2019-07-22

**Authors:** Xiaomei Liu, Biru Luo, Wentao Peng, Fei Xiong, Fan Yang, Jinhui Wu

**Affiliations:** 1 Pediatric Department of the West China Second Hospital of Sichuan University, Chengdu, China; 2 Nursing Department of the West China Second Hospital of Sichuan University, Chengdu, China; 3 Key Laboratory of Birth Defects and Related Diseases of Women and Children, Ministry of Education, Sichuan University, Chengdu, China

**Keywords:** Catch-up growth, Preterm infants, Discharged, Factors

## Abstract

**Background:**

The purpose of this study was to analyze the growth status and to identify the risk factors that influence the catch-up growth of preterm infants after discharge and to provide evidence for feeding strategies and the need for further research.

**Methods:**

A descriptive correlational analysis was applied. The sample consisted of 309 preterm infants and their caregivers selected from June to August 2017 from five women’s and children’s hospitals. Self-designed questionnaires based on knowledge, attitude and practice and the Health Belief Model (HBM) were used to measure the catch-up growth status of preterm infants after discharge. Logistic regression was used to determine the risk factors for the catch-up growth of preterm infants.

**Results:**

The results showed that of 309 preterm infants, only 14 (4.5%) were underweight, and 52 (17.4%) did not meet the criteria for catch-up growth at 12 months of actual age. The logistic regression analysis showed that gestational age, regular health care, caregivers’ educational background, mothers’ daily contact with the baby, monthly average family income, the addition of a breast milk supplement, and daily milk volume were risk factors that affected the catch-up growth of preterm infants after discharge.

**Conclusions:**

The rate of catch-up growth of preterm infants is still not high. We should pay much more attention to preterm infants of small gestational age and guide their child care on a regular basis to detect and correct risk factors in a timely fashion, especially those involving lower daily milk volume, lower degree of culture and family economic difficulties. Second, we suggest that the government publish relevant policy that appropriately increases the length of maternity leave for preterm mothers. Future studies should have larger sample sizes and explore other important factors influencing the catch-up growth of preterm infants.

## Background

With improvements in medical technology, the incidence and survival rate of preterm infants have increased substantially [1, 2]. However, many studies have also found that most preterm infants experience the problem of extrauterine growth retardation [3, 4]. Catch-up growth refers to growth retardation caused by pathological factors and the acceleration of growth after the removal of these factors. Studies have found that the catch-up growth of preterm infants is closely related to their motor and neurodevelopmental outcomes [5–7]. Therefore, it is important to study the risk factors for the catch-up growth of preterm infants after discharge to improve their long-term quality of life. Radmacher [8] found that the incidence of extrauterine growth retardation was highly correlated with birth weight. Some studies have noted that the factors affecting the growth of preterm infants include gestational age, birth weight, nutrition during hospitalization, degree of disease, and growth status before discharge [9]. The Health Belief Model (HBM) mainly analyzes the mechanism of behavior change from the perspective of attitudes and beliefs. Many studies have found that health beliefs could influence behavioral changes and improve disease outcomes [10, 11]. Health beliefs may affect caregivers’ feeding behaviors, thus affecting the catch-up growth of the preterm infants. The present study used the HBM to design questionnaires, and multicenter studies were conducted to investigate the factors influencing catch-up growth in preterm infants after discharge.

## Methods

### Materials

This multicenter study was conducted in Chengdu, China. A total of 309 preterm infants and their caregivers were selected from the Child Health Care Departments of 5 Women’s and Children’s Hospitals between June and August 2017. The inclusion criteria included the following: preterm infants between 1 and 3.5 years of age, voluntary participation in the study, and caregivers with normal language expression and reading comprehension ability. The exclusion criteria included the following: infants with congenital malformations and with severe disease, such as cerebral palsy, metastatic liver disease, chronic nephritis, and leukemia; and uncooperative caregivers. The criteria for elimination from the study included participant withdrawal from the study, which accounted for up to 20% of the missing data values.

### Data collection

We designed questionnaires based on the theory of the HBM. The social-demographic data of the preterm infants and their caregivers were collected, including information on sex, birth weight, gestational age, admission to the PICU or regular health care, and caregivers’ educational background and occupation type. We investigated the levels of the caregivers’ knowledge about feeding preterm infants after discharge, including information related to dairy choice, reasonable standards and importance of catch-up growth, knowledge of complementary foods and vitamin D addition. There were 18 items in total, with choices of “yes” and “no”; correct answers were scored as 1, and incorrect answers were scored as 0. The total score was 18 points, and a higher score indicated that the caregivers had better knowledge of the feeding of premature infants after discharge. The content validity of this questionnaire was evaluated by 6 experts, and the CVI value was 0.989. The reliability of the questionnaire was assessed by SPSS16.0, and Cronbach’s alpha was 0.713. We also investigated caregivers’ health beliefs, including their personal health beliefs, ability to implement, ability to control, belief in their ability to use resources, and cognitive threat. All results were scored with a five-point scale, ranging from 1 to 5 points. Higher scores indicate better health beliefs. The content validity of this questionnaire was evaluated by 6 experts, and the CVI value was 0.979. The reliability of the questionnaire was assessed by SPSS16.0, and Cronbach’s alpha was 0.911.

In addition, weight was measured by trained and tested professional surveyors and was accurate to 0.01 kg; we collected the preterm infants’ weight data at the actual age of 12 months, based on hospital records. A total of 368 questionnaires were issued, and 355 questionnaires were recovered, 309 of which were valid; thus, the effective rate was 87%.

### Definition of underweight and catch-up growth

Underweight was defined as a body weight below the 3rd percentile (P3^rd^) level [12] of the corrected age of the standard for children in nine cities in China [13]. Catch-up growth was defined as a body weight reaching the 10th percentile (P10^th^) of the corrected age for the small-for-gestational-age(SGA) children and the 25th percentile (P25^th^) of the corrected age for the appropriate-for-gestational-age (AGA) children [9]. The body length and head circumference measurement data were not analyzed due to wide variations in values and large amounts of missing data during data collection.

### Statistical analysis

To calculate the sample size, we used the following formula:

$$ n={\left(\frac{Z_{\alpha /2}}{\delta}\right)}^2\cdot \pi \left(1-\pi \right) $$

The allowable error for the rate of preterm infants was 5% with an overall rate of 7.8%, which meant that we needed 307 preterm infants and their caregivers [9]. The survey data were entered into Epidata 3.1, and we used SPSS16.0 for statistical analyses. Logistic regression analysis was used in this study to determine the risk factors for catch-up growth in preterm infants.

### Ethical consideration

The study was approved by the Ethics Committee of West China Second Hospital, Sichuan University. Informed consent was obtained from all participants.

## Results

### General situations of preterm infants and their caregivers

The results showed that the ratios of the birth weight were as follows: over 2000 g, 67.6% (209); 1500~2000 g, 21.7% (67); and below 1500 g, 10.7% (33). In addition, the ratios of the gestational age were as follows: over 34w, 67.3% (208); 32~34w, 19.1% (59); and below 32w, 13.6% (42). SGA vs AGA was (19.4% vs 80.6%), and cesarean vs natural was (70.6% vs 29.4%). Regarding fetus number, singles comprised 64.1% (198), doubles 35.3% (109), three and above 0.6% (2). Regarding parity, 79.9% (247) were first pregnancies, 17.8% (55) were second, and 2.3% (7) were third and above. There was no difference in the sex ratio (male vs female was 50.2% vs 49.8%).

### Possible related factors for catch-up growth in preterm infants after discharge

We considered many possible related factors in this study. Most of the preterm infants were admitted to the PICU before (75.4%) and had regular health care (93.5%). Nineteen caregivers had junior middle school education and below, 55 had senior middle school education, 87 had junior college education, 115 had undergraduate education, and 33 had graduate-level education and above. Mothers’ daily contact with the baby (< 10 h vs ≥10 h) was 27.5% vs 72.5%. Most of the feeding programs were made by the mother (80.3%), some were made by the older relatives (14.2%), and the remaining ones were the father and others (2.6 and 2.9%). The ratios of monthly average family income (RMB) were as follows: below 5000 (8.7%), 5000–10000 (40.5%), 10001–20000 (33.3%), and over 20000 (17.4%). There was no difference in the maternal history (Yes vs No was 155 vs 154). In addition, most of the preterm infants were breastfed (80.5%), and some of them had the addition of breast milk supplements (27.8%). The ratios of daily milk volume were as follows: lower than normal (10.4%), essentially normal (87.4%), and higher than normal (2.2%); furthermore, most of the premature infants had problems that required adding supplementary food (81.4%) and were previously diagnosed with malnutrition (71.2%).

### Postdischarge feeding knowledge scores of the preterm infants’ caregivers

The average total score of the postdischarge feeding knowledge of the preterm infants’ caregivers was 14.22 ± 2.42 points. The highest average score of all dimensions was for dairy selection, while the lowest was for vitamin D addition. These results are shown in Table 1.

**Table 1:** Postdischarge feeding knowledge scores of preterm infants’ caregivers

Dimensionality	Range	Mean ± standard deviation	Item average ± standard deviation
Dairy choice	0–5	4.22 ± 0.96	0.84 ± 0.19
Reasonable standards and importance of catch-up growth	0–3	2.34 ± 0.84	0.78 ± 0.28
Knowledge of complementary foods	0–8	6.07 ± 1.32	0.76 ± 0.16
Vitamin D addition	0–2	1.59 ± 0.64	0.8 ± 0.32
Total points	0–18	14.22 ± 2.42	0.79 ± 0.13

### Postdischarge feeding-related health beliefs of the preterm infants’ caregivers

The total score of the postdischarge feeding-related health beliefs of the preterm infants’ caregivers was 111.11 ± 12.53 points, but the average item scores for ability to control, belief in one’s ability to use available resources, ability to implement, and cognitive threat were all less than 4 points. The results are shown in Table 2.

**Table 2:** The postdischarge feeding-related health belief scores of preterm infants’ caregivers

Dimensionality	Range	Mean ± standard deviation	Item average ± standard deviation
Personal health beliefs	0–45	36.50 ± 4.44	4.06 ± 0.49
Ability to implement	0–15	10.99 ± 1.95	3.66 ± 0.65
Ability to control	0–30	23.40 ± 3.20	3.9 ± 0.53
Belief in the ability to use resources	0–40	29.98 ± 5.02	3.75 ± 0.63
Cognitive threat	0–15	10.26 ± 2.11	3.42 ± 0.7
Total points	0–145	111.11 ± 12.53	3.7 ± 0.42

### Conditions of catch-up growth in preterm infants after discharge

We compared weight in infants with an actual age of 12 months with the corresponding corrected age standard weight to determine whether the criterion of catch-up growth was achieved. The results are shown in Table 3.

**Table 3:** The condition of infants with underweight and catch-up growth at 12 months of actual age (*n* = 309)

Type	Underweight		Catch-up growth	
	Yes	No	Yes	No
SGA	6 (1.9%)	54 (17.5%)	53 (17.2%)	7 (2.3%)
AGA	8 (2.6%)	237 (76.7%)	218 (70.6%)	31 (10.0%)

### Multifactor analysis of the factors affecting the catch-up growth of preterm infants after discharge

We considered the criterion of catch-up growth as the dependent variable and the possible related factors as independent variables. Multivariate logistic regression analysis was performed. The inclusion level was 0.05 (α in = 0.05), and the elimination level was 0.10 (α out =0.10). The result of the goodness-of-fit test using the Hosmer-Lemeshow method was χ2 = 9.908, ν = 8, *P* = 0.272, and the fitting rate was 96.7%. The determinant coefficient of the regression model R^2^ was 0.328. It was found that gestational age, regular health care, mothers’ daily contact with the baby, caregivers’ educational background, monthly average family income, the addition of breast milk supplements and daily milk volume were the risk factors for the catch-up growth of preterm infants after discharge. The results are shown in Table 4.

**Table 4:** Logistic regression analysis on factors influencing the catch-up growth of preterm infants after discharge

Variable	Partial regression coefficient	Standard error	Wald	*P*-value	OR (95%CI)
Birth weight	−1.248	0.663	3.548	0.060	0.287 (0.078–1.052)
Gestational age	1.801	0.664	7.351	0.007	6.055 (1.647–22.259)
Type	0.889	0.805	1.219	0.269	2.433 (0.502–11.788)
Delivery mode	−0.899	0.563	2.544	0.111	0.407 (0.135–1.228)
Number of fetuses	0.923	0.571	2.613	0.106	2.518 (0.822–7.715)
Parity	−0.670	0.606	1.222	0.269	0.512 (0.156–1.678)
Admission to the PICU	0.870	0.518	2.816	0.093	2.386 (0.864–6.588)
Regular health care	1.545	0.757	4.166	0.041	4.687 (1.063–20.656)
Caregivers’ educational background	−0.532	0.254	4.395	0.036	0.587 (0.357–0.966)
Mothers’ daily contact with the baby	2.305	0.823	7.838	0.005	10.029 (1.997–50.372)
Head of the feeding program	−0.636	0.735	0.750	0.386	0.529 (0.125–2.234)
Monthly average family income	0.817	0.306	7.113	0.008	2.264 (1.242–4.128)
Maternal history	0.805	0.502	2.579	0.108	2.238 (0.837–5.98)
Breastfeeding	−0.092	0.636	0.021	0.885	0.912 (0.262–3.174)
Addition of breast milk supplements	1.453	0.676	4.620	0.032	4.274 (1.137–16.071)
Daily milk volume	1.362	0.557	5.978	0.014	3.904 (1.31–11.633)
Problem with adding supplementary food	0.497	0.577	0.741	0.389	1.643 (0.531–5.089)
Previously diagnosed with malnutrition before	−0.766	0.527	2.108	0.147	0.465 (0.165–1.307)
Knowledge score	0.024	0.087	0.076	0.783	1.024 (0.863–1.216)
Health belief score	0.027	0.021	1.605	0.205	1.027 (0.985–1.071)

Birth weight: < 1500 g = 1, 1500~2000 g = 2, > 2000 g = 3; Gestational age: <32w = 1, 32~34w = 2, > 34w = 3; Type: SGA = 1,AGA = 2; Delivery mode: Caesarean = 1, Natural = 2; Number of fetuses: Single = 1, Double = 2, Three and above = 3; Parity: First = 1, Second = 2, Other = 3; Admission to the PICU: Yes = 1,No = 2; Regular health care: No = 1,Yes = 2; Caregivers’ educational background: Junior middle school and below = 1, Senior middle school = 2, Junior college = 3, Undergraduate = 4, Graduate students and above = 5; Mothers’ daily contact with the baby: <10 h = 1, ≥ 10 h = 2,; Head of the feeding program: Mother = 1, Father = 2, Ancestors = 3, Other = 4; Monthly average family income (RMB): <5000 = 1, 5000–10000 = 2, 10001–20000 = 3, >20000 = 4; Maternal history: Yes = 1, No = 2; Breastfeeding: Yes = 1, No = 2; Addition of breast milk supplements: No = 1,Yes = 2; Daily milk volume: Lower than normal = 1, Basically normal = 2, Higher than normal =3; Problem with adding supplementary food:Yes = 1,No = 2; Previously diagnosed with malnutrition before:Yes = 1, No = 2

## Discussion

The results showed that only 14 of the 309 preterm infants (4.5%) were underweight at 12 months of actual age. The incidence was lower than those of Sharma’s [14] and Mukhopadhyay’s [15] studies, which may be due to the higher proportion of extremely low-birth-weight premature infants (30 and 15%, respectively) in their studies; in contrast, only 4 infants (1.3%) in this study were considered to have extremely low birth weight. Second, the incidence was also lower than that found in the results of Deng Ying’s survey in China [16]. It may be that only the very-low-birth-weight infants were included in that survey, but very-low-birth-weight infants comprised only 10.7% of the sample in this study. However, the study found that 52 infants (17.4%) did not meet the criteria for catch-up growth at 12 months of actual age. It can be seen that the incidence of catch-up growth in preterm infants was still not high. Current studies have shown that catch-up growth affects physical [17], nerve and motor development. Therefore, it is still necessary to pay more attention to catch-up growth in preterm infants after discharge and to help them achieve satisfactory catch-up growth to improve their long-term quality of life.

The logistic regression analysis showed that gestational age, regular health care, caregivers’ educational background, mothers’ daily contact with the baby, monthly average family income, the addition of breast milk supplement and daily milk volume were factors that affected the catch-up growth of preterm infants after discharge.

First, our study found that a smaller gestational age increased the risk of catch-up growth. Gestational age is closely related to the incidence of complications in premature infants. With the increase in gestational age, the incidence of various complications in preterm infants will gradually be lower, thus facilitating a more successful feeding process and the completion of catch-up growth. Bjelanovic V et al. [18] found that the smaller the gestational age was, the more complications preterm infants had. According to Clark’s [3] research, with decreases in gestational age and birth weight, the incidence of EUGR for each parameter of premature weight, body length and head circumference will increase. Therefore, preterm infants of small gestational ages merit much more monitoring after discharge.

We found that preterm infants receiving health care services on a regular basis were 4.687 times more likely to achieve catch-up growth than were preterm infants receiving health care services on an irregular basis. It is possible that among preterm infants receiving regular health care, medical personnel could find deviations in the growth of premature infants in a timely manner and could give caregivers more feeding guidance and control measures; in this way, the premature catch-up growth situation was relatively good. Therefore, it is recommended that preterm infants receive health care regularly.

In addition, we found that caregivers’ educational background, mothers’ daily contact with the baby and monthly average family income were also factors affecting catch-up growth; this finding may have arisen because the higher the caregivers’ educational background is, the longer the time of contact between the mother and the baby. Additionally, a higher monthly average family income would facilitate access to more comprehensive care for preterm infants and provide more material comfort for them, such as breast milk supplements and preterm infant formula; thus, the catch-up growth was also better.

Furthermore, the results showed that the addition of breast milk supplementation and daily milk volume were factors that affected the catch-up growth of preterm infants after discharge. Breastfeeding has many benefits, but its exclusive use in certain situations can lead to nutrient deficiencies and bone demineralization [19, 20], especially for very and extremely low-birth-weight infants. It has been recognized internationally that fortified breast milk is the best choice of nutrition for preterm infants at present, to meet the baby’s protein, energy and vitamin needs for their catch-up growth [9, 21]. However, there is some controversy about the results of studies on the effect of breastfeeding on premature infants. Some studies showed that the weight, body length and head circumference in the fortified breastfeeding group were superior to those of the breastfeeding group at 3 months of corrected age, but there was no statistically significant difference in the long-term neural development assessment and the growth index between the two groups [22–24]. Furthermore, the current research sample size of breast milk supplements was small; in the future, the sample size should be increased with multicenter research. In addition, we found clinically that preterm infants with more milk per day received more nutrition and more readily experienced catch-up growth. Premature babies are generally recommended to consume 110~130 kcal/kg every day. This study was a retrospective study, which could not accurately define the standard of daily milk grading. The standard should be improved in future studies.

## Conclusion

In summary, preterm infants of small gestational ages should be closely monitored after discharge. Their parents should be advised to take their children to receive health care services regularly to allow for risk factors to be detected and addressed in a timely manner, especially the factors pertaining to lower daily milk volume, caregivers with lower educational backgrounds and families with difficult economic circumstances. Second, we suggest that the government publish relevant policy that appropriately increases the length of maternity leave for mothers of preterm infants. In this way, mothers will be able to maintain contact with their children for a much longer period of time and thus smoothly promote catch-up growth, improve the quality of long-term survival, and reduce the burden on society.

More physical index data can be added in the future for analysis and judgment, such as information on height, head circumference and body mass index (BMI), to more comprehensively evaluate the completion status of catch-up growth of preterm infants after discharge.

In addition, it is important to note that previous health belief-related studies have shown that health beliefs change the objectives of the study of health behavior, but this research shows that the health beliefs of caregivers had no significant effect on the catch-up growth of preterm infants. Moreover, the determination coefficient R^2^ of this regression model is 0.328, indicating that there are still many as-yet-unidentified factors influencing the catch-up growth of preterm infants. Therefore, future studies should have larger sample sizes and explore other important factors influencing the catch-up growth of preterm infants.

## Data Availability

The datasets used and/or analyzed during the current study are available from the corresponding author on reasonable request.

## Abbreviations

BMI: Body Mass Index
HBM: Health Belief Model
